# Does Total Wrist Arthroplasty for Treatment of Posttraumatic Wrist Joint Osteoarthritis in Young Patients Always Lead to Restriction of High-demand Activities of Daily Living? Case Report and Brief Review of Recent Literature

**DOI:** 10.2174/1874325001711010439

**Published:** 2017-05-30

**Authors:** Ingo Schmidt

**Affiliations:** SRH Poliklinik Gera GmbH, Straße des Friedens 122, 07548 Gera, Germany

**Keywords:** Wrist, Radiocarpal dislocation, Greater arc injury, Posttraumatic ulnar carpal translocation, Posttraumatic pancarpal wrist joint osteoarthritis, Total wrist arthroplasty

## Abstract

**Background:**

Posttraumatic ulnar carpal translocation is a very rare condition that is caused either by fracture-dislocation injury or by purely ligamentous injury of the wrist. Its prognosis is poor and development of posttraumatic pancarpal wrist joint osteoarthritis is inevitable, and options for treatment are total wrist fusion or total wrist arthroplasty.

**Methods:**

A 24-year-old male sustained a fracture-related injury in his left wrist that was accompanied with a second ligamentous distorsion-related injury 1 year later in the same wrist. Seven years after first injury, a posttraumatic pancarpal wrist joint osteoarthritis has developed that was caused by posttraumatic ulnar carpal translocation. The patient was treated by total wrist arthroplasty with use of the Maestro^TM^ Wrist Reconstructive System.

**Results:**

With our patient, it is unclear whether posttraumatic ulnar carpal translocation occurred either as result of the first fracture-related injury or as result of the second ligamentous distorsion-related injury or as result of both injuries. The 31-year-old patient could be reemployed completely in his original occupation as a mechanic for big agriculture machines and load his wrist with more than 10 pounds. In order to preserve motion, the patient reported that he would undergo the same total wrist arthroplasty a second time were it necessary.

**Conclusion:**

We report on a young male receiving total wrist arthroplasty and resulting in good restoration of his high-demand claims in activities of daily living, respectively. However, it cannot be concluded that total wrist arthroplasty is to be preferred generally over total wrist fusion in young patients. Essential prerequisite for this motion-preserving procedure is the compliance of patients.

## INTRODUCTION

Posttraumatic ulnar carpal translocation (PUCT) is a very rare condition that is caused either by fracture-dislocation injury or by purely ligamentous injury of the wrist mainly involving the volar extrinsic radioscaphocapitate ligament (RSCL). The diagnosis of PUCT is often delayed, and the outcomes after surgical repair are poor potentially leading to posttraumatic pancarpal wrist joint osteoarthritis (OA). For treatment of posttraumatic pancarpal wrist joint OA there are 2 options: a total wrist fusion (TWF) or a motion-preserving total wrist arthroplasty (TWA). Both of these procedures have advantages and disadvantages. In decision making which procedure is to be preferred, the age of patients and their claims in activities of daily living have to be considered. Currently, two questions are not clearly answered: first, how can patients load their wrists with a TWA; and second, are young patients suitable receiving a TWA. First, we report on a 24-year-old male who sustained a possible fracture-dislocation injury in his left wrist, and 1 year later an additional purely ligamentous injury in the same wrist that led to PUCT in a course over 7 years and finally resulted in a TWA with patient's age of 31 years. The patient could be reemployed in his original occupation as a mechanic for agriculture machines and load his wrist with more than 10 pounds. Second, a brief review of literature on PUCT and recent evidence of TWA will highlight the case presentation.

## CASE PRESENTATION

**Fig. (1):**
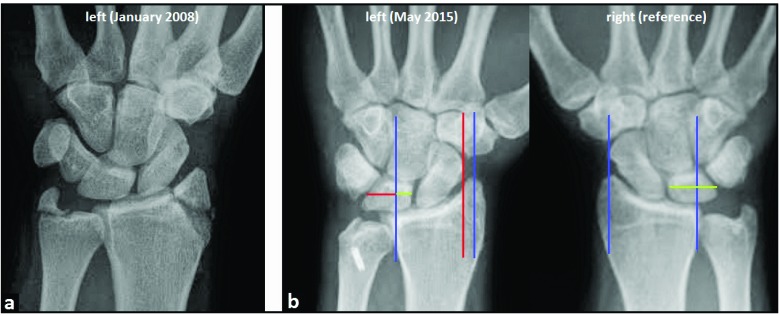
**(Case Presentation, preoperative radiographic course over 7 years in comparison to the uninjured right wrist as reference: blue longitudinal and green transverse lines): (a)** PA radiograph of the left wrist showing primary fractures of the radial and ulnar styloid without SLL disruption and without PUCT; **(b)** PA radiograph of the left wrist 7 years after primary injury showing PUCT type I involving the entire carpus (red longitudinal and transverse lines) in comparison to the uninjured right wrist, less than 50% of the left lunate bone articulates in its fossa of distal radius whereas more than 50% of the right lunate bone articulates with the opposing bone, there is no positive ring sign in left scaphoid bone detecting scapholunate instability, note that the 2 screws for ORIF of radial and ulnar styloid were formerly removed.

**Fig. (2):**
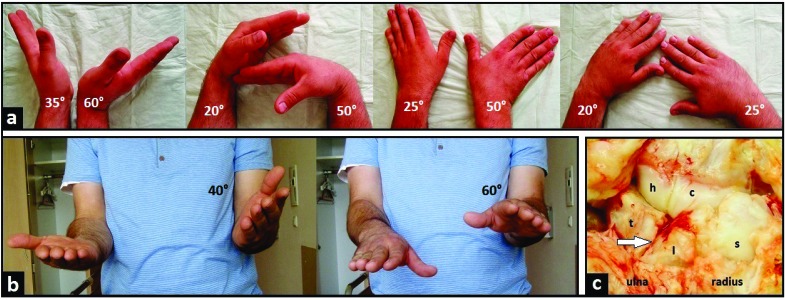
**(Case Presentation, pre- and intraoperative findings): (a)** Clinical photographs demonstrating impaired wrist joint motion in comparison to the uninjured right wrist; **(b)** Clinical photographs demonstrating impaired supination and pronation in comparison to the uninjured right wrist; **(c)** Intraoperative clinical photograph demonstrating posttraumatic pancarpal wrist joint OA, note the disruption of LTL (arrow) in the absence of disruption of SLL that confirmed the preoperative radiographic finding of PUCT type I.

**Fig. (3):**
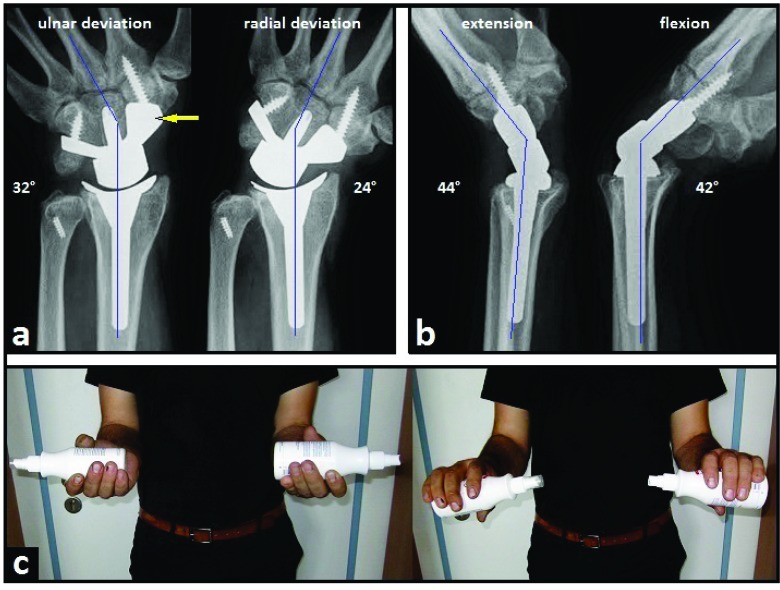
**(Case Presentation, 1-year follow-up): (a)** PA radiographs in terms of terminal ranges of motion showing unchanged correct positioning of TWA without implant loosening or subsidence or impingement or instability, note that the entire scaphoid bone was excised and a carpal plate utilizing a scaphoid augment (arrow) was used that allows its sufficient support onto the base of trapez bone without the necessity of fusion between the distal part of scaphoid bone to the surrounding carpal bones; **(b)** Lateral radiographs in terms of terminal ranges of motion showing no instability of implant; **(c)** Clinical photographs demonstrating 90° supination and 90° pronation of both forearms.

**Fig. (4):**
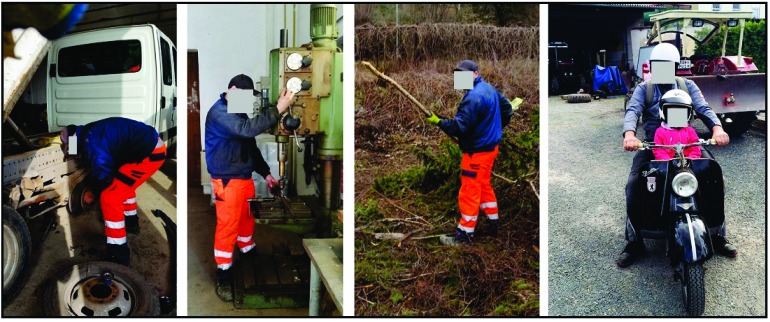
**(Case Presentation, 16-months follow-up):** Self made photographs by the patient demonstrating complete reemployment in his original occupation and leisure, note that the patient loads his left wrist with the TWA more than 10 pounds.

A 24-year-old and right-hand-dominant male presented with left intraarticular displaced radial styloid fracture accompanied with an ulnar styloid base fracture after a fall on his left wrist (Fig. ** 1**). It was treated surgically with open reduction and internal fixation (ORIF) using 2 screws in another hospital, and the patient could be reemployed pain-free in his original occupation as a mechanic for agriculture machines. One year after the first injury, the patient sustained a second injury in his left wrist without fracture, diagnosed as a ligamentous distorsion. Two years after the first injury, the screw for reduction of the ulnar styloid has been broken due to a non-union, and all screws were removed in the same hospital. Four years after the first injury, the patient was unable to work in his original occupation, and presented himself for the first time in our hospital. The non-unioned ulnar styloid was excised accompanied with surgical denervation of the wrist. After that, the patient could be reemployed in his original occupation for another three years. Then, the 31-year-old patient was unable to work in his original occupation again. Radiographically, there was advanced stage of posttraumatic wrist joint OA due to a distinctive PUCT type I without signs of disruption of the scapholunate ligament (SLL) (Fig. **1b**), accompanied with marked loss of wrist and forearm function (Figs. **2a**-**b**). The patient declined a TWF, and a TWA using the relatively new Maestro^TM^ Wrist Reconstructive System ((WRS), (Zimmer Biomet Holdings, Warsaw, Indiana / USA)) was detected by us. Intraoperatively, macroscopic findings confirmed pancarpal wrist joint OA in the absence of a SLL disruption, whereas a disruption of the lunotriquetral ligament (LTL) was present (Fig. **2c**). At the 1-year follow-up, there was unchanged correct positioning of TWA without implant loosening or subsidence, and an excellent functional outcome without any signs of instability in terms of terminal ranges of motion radiographically (Fig. **3**). Supination and pronation had improved to 90° (100% to right), extension had improved to 44° (73,3% to right), flexion had improved to 42° (84% to right), ulnar deviation had improved to 32° (64% to right), and radial deviation had improved to 24° (96% to right) (Figs. **3a**-**c**) in comparison to (Figs. **2a**-**b**). At the 16-months follow-up, the patient could be reemployed completely in his original occupation as a mechanic for agriculture machines, and he is able to carry out heavy works with loads more than 10 pounds again (Fig. **4**). Pain in visual analogue score (scale 0-10 points) and function in patient-rated wrist evaluation score (scale 0-100 points) had improved to 2 (preop. 9) and 19 (preop. 68). The prolonged time of 16 months for recovery in our patient was not caused by the TWA, there were other difficulties in his life. The patient reported that he would undergo the same motion-preserving TWA a second time were it necessary.

## DISCUSSION

It is unclear with our patient whether PUCT occurred either as a result of the first fracture-related injury or as result of the second ligamentous distorsion-related injury or as result of both injuries. The problem is that the patient could no longer give any more details about the 2 injuries at time of first presentation in our hospital. First, PUCT of the wrist without accompanying fractures of the distal radius, distal ulna, and/or carpal bones, is uncommon and was first described in 1930 by Lorenz Böhler (†1973) [1]. PUCT is 1 of the 3 major proximal carpal instabilities and the injury pattern typically resulting from high-energy forces through the proximal arc of the wrist [2, 3]. The mechanism of injury appears to be hyperextension of the wrist, combined with a torque and pronation of the wrist or forearm, and/or ulnar deviation on a fixed hand in which most of all volar extrinsic radiocarpal ligaments, especially the stout RSCL and the long radiolunate ligament are ruptured [2-6]. Two types have been described by Taleisnik [3]: type I is associated with ulnar translocation of the entire carpus such as in our case, and type II is associated with SLL disruption and the scaphoid bone is not involved in ulnar translocation. Second, it also can be suggested that our patient sustained primarily a traumatic radiocarpal dislocation with disruption of the extrinsic RSCL, and the injury may be concealed by spontaneous reduction or inadvertently reduced with gentle manipulation, followed by progredient PUCT type I. Dumontier *et al.* [7] classified these dislocations into two types: type I includes purely ligamentous injuries with or without a small radial styloid avulsion associated with a high risk of persistent radiocarpal instability and generally poor outcomes if they present with PUCT, and type II includes a radial styloid fracture involving at least one-third of the scaphoid fossa associated with good outcomes if they can be reducted anatomically. However, despite primary surgical repair of traumatic radiocarpal dislocations, PUCT was observed in 23% of cases at an average follow-up of 2,8 years [8]. Herzberg *et al.* [9] have reported that the diagnosis of wrist fracture-dislocations (greater arc injuries), first described in 1855 by Joseph-François Malgaigne († 1865), was missed initially in 25% of cases. When using magnet resonance arthrography in patients with persistent posttraumatic wrist pain, a disruption of the extrinsic RSCL was found in 13,9% of 72 cases respectively, and a statistically significant (p < 0,001) correlation with concomitant disruptions of the intrinsic SLL and/or LTL has been observed as well [10]. Such as in our case, disruptions of LTL usually show no radiographic abnormality, only in severe cases the wrist will assume a volar intercalated segment instability with volar angulation of the lunate bone [11].

Due to the unfamiliarity of PUCT by treating physicians, the diagnosis is often delayed up to averaged 7, 3 months, and it may be present when less than 50% of the lunate bone articulates with the radius in neutral position in the posteroanterior (PA) radiograph [12-14]. Nonetheless, all measurements are quite variable and should be compared to radiographs of the opposite uninjured wrist [15]. Surgical treatments include repair of radiocarpal ligaments, tendon augmentation, radiocarpal pinning or external fixation. However, persistent or recurrent PUCT within the first postoperative year associated with patient's disability and late arthritis have been recognized in nearly all cases, regardless of the repair technique or surgical timing, and early radiolunate fusion to prevent recurrence was recommended by Rayhack *et al.* [12]. However, fusions crossing the radiocarpal row cause a loss of approximately 55% of the pre-fusion flexion-extension range of motion [16]. Posttraumatic degenerative changes in the wrist have been described in majority of all cases at average follow-up's ranging from 32 months to 6,5 years, despite primary surgical restoration and/or stabilization of radiocarpal alignment [12, 17]. Irreparable cartilage damage may have occurred at the time of initial injury from compression, shear, and torsional forces. In addition, abnormal wrist mechanics in PUCT may have contributed to joint deterioration over time [17].

Recently, rheumatoid arthritis remains the most common indication for TWA with relative portion ranging from 51-71% of all patients receiving TWA [18, 19]. However, TWA has proven to be useful as a motion-preserving alternative to TWF for treatment of posttraumatic wrist joint OA as well, and also resulted in a significantly better outcome than in patients who underwent a primary TWF [20, 21]. TWA for treatment of posttraumatic wrist joint OA (including scaphoid non-union/scapholunate advanced collapse) has reported to be a relative portion of 14% of all TWAs performed by surgeons who have published their experiences with this procedure [18]. The relatively new angle-stable Maestro^TM^ WRS that was used in our case is one of the modern biaxial-anatomical third generation type that is currently in use [22-24]. In a single-center study, published in 2015, the cumulative implant survival after eight years (N=68) is reported to be 95%, and at the 5-year follow-up radiographic loosening was present in 2% of all cases only [25]. Currently, the Maestro™ implant achieves the most favorable functional outcome as compared to other third-generation types, and it may be justified in preserving resection-related carpal height due to its three various carpal heads in combination with its design of ellipsoid surface articulation [26-28]. Additionally, the design of the Maestro™ implant allows the combination with an ulnar head prosthesis and/or a thumb carpometacarpal total joint replacement [29, 30]. Recently, implant survival with the third TWA generation types is reported to be 90-100% at 5 years in most series, but it declines from 5 to 8 years [18]. Hence, patients undergoing treatment with TWA must be prepared that it might end with TWF; therefore limited bone resection is a common feature of all contemporary wrist replacements [31, 32]. The Maestro™ WRS has demonstrated uncomplicated conversion to TWF [25, 26, 33, 34]. A recent study revealed that the outcome of TWF after a failed TWA is comparable to those after primary TWF [35].

Two questions are not clearly answered currently: first, how can patients load their wrists with a TWA; and second, are young patients suitable receiving a TWA. In the past, TWA was almost exclusively used in older patients with rheumatoid arthritis because of their low-demand lifestyle, whereas patients with posttraumatic OA are typically young and active [36, 37]. One major complication with the use of older TWA generation types was instability or dislocation in up to 8% of cases [18]. It has been noted by Sterling Bunnel (†1957): “A painless stable wrist is the key to hand function” [38]. This problem seems to be solved with the use of the new third generation types. The rates of instability or dislocation for the RE-MOTION^TM^ total wrist is reported to be 0,7% only (1 of 144 cases) [18], and for the Maestro™ WRS 0,6% only (1 of 157 cases) [25, 26, 34, 39, 40]. Recently, the age of patients for whom a TWA can be indicated is not always focused for the elderly, the youngest patients receiving total wrist joint replacements were reported to be 25 and 31 years old [26, 41]. In the literature, no evident data exist on how patients can load their wrists with a TWA. Weiss and Akelman [42] advised their patients not to load the wrists greater than 10 pounds which contains the total wrist implant from a safety perspective. On the other hand, Nicoloff [43] and Schmidt [44] reported on 2 cases in which the patients with a TWA could be reemployed successfully in their original occupations as an engineering worker under earth and a tiler, both patients load their wrists more than 10 pounds.

## CONCLUSION

We report on a young male receiving TWA for treatment of posttraumatic wrist joint OA after a rare PUCT that results in good restoration of his high-demand claims in activities of daily living, respectively. However, it is not the intention of this article to advocate for general use of TWA in young patients. Essential prerequisite for such a motion-preserving procedure is the compliance of patients. In conclusion, Herzberg and Adams [45] noted in 2012: “The future appears positive for greater adoption of total wrist arthroplasty for patients who are willing to accept somewhat greater risks for the benefit of improved function”. Recent evidence using the National Inpatient Sample database (USA) suggests that the complication rate of TWA with 10% does not differ significantly to those with 7% in patients undergoing a TWF [19]. Also, it is noted that all surgeons who are performing TWAs need a learning curve for realization of correct insertion of an implant [46].

## LIST OF ABBREVIATIONS

LTL: = Lunotriquetral ligament
OA: = Osteoarthritis
ORIF: = Open reduction and internal fixation
PUCT: = Posttraumatic ulnar carpal translocation
RSCL: = Radioscaphocapitate ligament
SLL: = Scapholunate ligament
TWA: = Total wrist arthroplasty
TWF: = Total wrist fusion
WRS: = Wrist Reconstructive System
